# Proteomic Analysis of *Rhizoctonia solani* Identifies Infection-specific, Redox Associated Proteins and Insight into Adaptation to Different Plant Hosts* [Fn FN2]

**DOI:** 10.1074/mcp.M115.054502

**Published:** 2016-01-25

**Authors:** Jonathan P. Anderson, James K. Hane, Thomas Stoll, Nicholas Pain, Marcus L. Hastie, Parwinder Kaur, Christine Hoogland, Jeffrey J. Gorman, Karam B. Singh

**Affiliations:** From the ‡CSIRO Agriculture, Floreat, Western Australia;; §The University of Western Australia Institute of Agriculture, Crawley, Western Australia;; ¶QIMR Berghofer Medical Research Institute, Herston, QLD, Australia

## Abstract

*Rhizoctonia solani* is an important root infecting pathogen of a range of food staples worldwide including wheat, rice, maize, soybean, potato and others. Conventional resistance breeding strategies are hindered by the absence of tractable genetic resistance in any crop host. Understanding the biology and pathogenicity mechanisms of this fungus is important for addressing these disease issues, however, little is known about how *R. solani* causes disease. This study capitalizes on recent genomic studies by applying mass spectrometry based proteomics to identify soluble, membrane-bound and culture filtrate proteins produced under wheat infection and vegetative growth conditions. Many of the proteins found in the culture filtrate had predicted functions relating to modification of the plant cell wall, a major activity required for pathogenesis on the plant host, including a number found only under infection conditions. Other infection related proteins included a high proportion of proteins with redox associated functions and many novel proteins without functional classification. The majority of infection only proteins tested were confirmed to show transcript up-regulation during infection including a thaumatin which increased susceptibility to *R. solani* when expressed in *Nicotiana benthamiana*. In addition, analysis of expression during infection of different plant hosts highlighted how the infection strategy of this broad host range pathogen can be adapted to the particular host being encountered. Data are available via ProteomeXchange with identifier PXD002806.

The fungal pathogen of plants, *Rhizoctonia solani*, commonly causes disease by infecting roots and stems and inducing necrotic lesions from which it obtains nutrients. Isolates of the species cause major losses to the production of important crops including wheat, rice, soybean, maize, potato, sugarbeet, and many more (1, 2). It has been observed that plant cell death occurs in advance of the invading hyphae (3) suggesting the fungus produces mobile substances to diffuse and manipulate the host plant into susceptibility. How this is achieved is presently unknown, though some studies have identified phenolic compounds, phenyl acetic acid and derivatives or carbohydrate based host specific toxins in the rice-*R. solani* AG1–1A interaction. In addition, the specific down-regulation of defense genes in tobacco was observed following *R. solani* challenge and this could be circumvented by co-inoculation with the biocontrol fungus *Trichoderma harzianum* which provided enhanced resistance to *R. solani* (4). These findings suggest *R. solani* employs an active mechanism to suppress defenses and manipulate the host into susceptibility. In some plant-fungal pathosystems the conditioning the plant for susceptibility to the invading pathogen has been attributed to the production and secretion of proteinaceous toxins or effectors (5–7).

For biotrophic fungi, suppression of plant innate immunity is required to allow extended periods of habitation of plant tissue by the pathogen during which intimate feeding structures are produced allowing the pathogen to extract nutrients from living plant cells. Despite an absence of this close intracellular relationship with the host for many necrotrophic fungi, proteinaceous effectors have also been shown to play a role. For example *Botrytis cinerea* contains two NEP1 (necrosis and ethylene inducing protein)-like proteins (NLPs)^1^, which contribute to pathogenicity on a broad range of hosts (8). The NLPs are secreted phytotoxin proteins found in a phyllogenetically diverse range of necrotrophic pathogens and induce rapid tissue necrosis and plant immune responses which promote susceptibility to the infecting pathogen (9, 10).

Although some necrotrophic effectors act on a broad range of host plants, others appear to be more specific, mimicking effectors from biotrophic pathogens and interacting with specific R protein like receptors in the plant. Activation of the R proteins induces the plants defenses against biotrophs, including cell death, but in the case of necrotrophs this appears counterproductive and in turn enhances susceptibility to the pathogen. One example of this is the wheat tan spot fungus, *Parastagonospora nodorum*, which produces a variety of proteinacious host specific toxins including ToxA (11). ToxA is perceived by the wheat NBS-LRR protein Tsn1, which initiates host cell death and susceptibility (12). Another example appears to be the cyclic peptide effector victorin from *Cochliobolus victoriae.* Oat lines carrying the *R* gene *Pc2*, which otherwise provides resistance to the biotroph *Puccinia coronate*, recognize victorin and this leads to activation of host cell death and susceptibility to the necrotrophic pathogen (13). A similar situation occurs in Arabidopsis where it is hypothesized that victorin mimics a biotroph effector in order to be recognized by a NBS-LRR R protein, which induces the host cell death defense response and subsequently susceptibility to the pathogen (14).

Despite the large impact of diseases caused by *R. solani* on a range of economically important agricultural species, little is known about proteins contributing to pathogenesis in this species nor the components of effective defense in many of the broad range of host plant species (15, 16). Nonetheless, substantial insights have been made for several host-*R. solani* interactions. For example, analysis of differential gene expression in tobacco challenged with compatible or incompatible strains of *R. solani* identified a protein kinase gene, *NtPK*, which when over-expressed caused the up-regulation of defense associated genes and enhanced resistance to *R. solani*. Conversely, silencing the protein kinase reduced resistance and defense gene expression, suggesting the protein kinase plays a crucial role in effective defense of tobacco against *R. solani* (17). Recent advances in genome sequencing and comparative genomics of *R. solani* isolates (2, 18–20) have enabled prediction of genes putatively associated with pathogenesis from isolates with different hosts, however, further supportive evidence is required to identify those with a function in pathogenicity. One approach to this is through the identification of proteins produced and accumulated intracellularly or secreted during infection of the host plant. The close association between fungus and host add complexities of plant contamination of samples because the proportion of fungal tissue is low, particularly at the early stages of infection when effector functions are often most critical. Where protein information is used for annotation of gene structure in fungal genomes, this can be particularly problematic as host peptides with similarity to fungal sequences may guide incorrect annotation of fungal genes. We have therefore developed a technique to enable collection and mass spectrometry based proteomic analysis of samples highly enriched with fungal tissue and the surrounding culture filtrate during infection of wheat roots. Soluble and membrane proteins were collected separately from mycelia samples and extracellular proteins precipitated from culture filtrate. This approach enabled an in-depth analysis of proteome structure under different conditions and identified 96 proteins exported from the fungus exclusively during pathogenicity thereby providing clues to understand the pathogenicity mechanism of *R. solani*. Subsequent analysis of transcript abundance in infected wheat root tissue and vegetative grown mycelium supported the infection-related up-regulation of the majority of genes tested. Among the *R. solani* proteins identified were secreted proteins and several novel small cysteine rich proteins, cell wall degrading proteins and proteins with many redox related functions. Characterization of the expression of pectate lyase genes during infection of wheat and the legume, *Medicago truncatula*, suggests the infection strategy is optimized to the host being encountered and supports previous observations (2) that adaptation to broad host range in *R. solani* AG8 may have been accompanied by expansion of gene families to degrade a variety of cell wall types. Moreover, *in-planta* expression of a *R. solani* thaumatin gene identified from culture filtrate in the infection conditions increased susceptibility to *R. solani*, supporting the utility of this approach for uncovering novel genes contributing to the pathogenicity mechanisms of uncharacterized pathogens.

## EXPERIMENTAL PROCEDURES

### 

#### 

##### Fungal Isolate

*Rhizoctonia solani* isolate WAC10335 from anastomosis group 8 was initially isolated from lupin from Western Australia. Anastomosis group was confirmed by ribosomal ITS sequences and host-range was confirmed by inoculation assays on wheat, lupin, *Medicago truncatula* and Arabidopsis (21–23). A description and genome sequence of this isolate was recently published (2). The isolate was stored at −80 °C on millet seed and prior to use in experiments was grown on freshly prepared PDA plates and incubated at room temperature for 7 days.

##### Growth of the Fungus in Vegetative and Infection Conditions

Sterile nitrocellulose membrane (Millipore, Darmstadt, Germany) was place on to the surface of a PDA plate and a square of inoculum from a prior PDA culture of fungus was placed on top of the membrane. The fungus was allowed to grow at room temperature for 1 week until it had almost covered the surface of the 8.5 cm diameter circle of nitrocellulose. Wheat seeds were surface sterilized in sodium hypochlorite solution (1.7% available chlorine) with shaking for 15 min and rinsed 3 times in sterile water. The seeds were added to a Petri dish containing filter paper moistened with sterile water and transferred to 4 °C for 3 days, then transferred to 24 °C in the dark for 3 days to germinate. The seedlings were removed from the Petri dishes and added to fresh plates containing 15 ml of minimal medium. The nitrocellulose membrane and attached mycelium was removed from the PDA plate and placed mycelium side down onto the wheat seedlings in minimal medium. The plates were incubated at 24 °C for 3 days (early time point) or 7 days (late time point) with 16 h of light (100 μm.m^−1^·s-^1^) prior to harvesting. The membrane and attached mycelium was removed from the plates, leaving behind the wheat seedlings. The membrane was rinsed with sterile water, the mycelium peeled from the membrane, blotted dry and frozen in liquid nitrogen. The culture filtrate from the infection was collected, centrifuged at 6000 × *g* for 15 min and filter sterilized. The wheat seedlings were rinsed and frozen in liquid nitrogen. Vegetative fungal samples were treated as above with the omission of wheat seedlings. The 3-day postinoculation vegetative culture filtrate samples had very low protein concentration and thus were not further analyzed. Five replicates for each sample type were pooled for protein extraction and analysis.

##### Protein Isolation

Mycellium was ground to a fine powder while frozen and an aliquot taken for membrane protein extraction and another for soluble protein extraction. Membrane protein was extracted using a Mem-PER Plus Membrane Protein Extraction Kit (Thermo Scientific, Waltham, MA) following the manufacturer's instructions. Soluble proteins were extracted by suspension of the ground mycelium in 5 volumes of 10% (w/v) trichloroacetic acid, 0.07% (v/v) 2-mercaptoethanol in acetone and incubation at minus 20 °C over night. Proteins were collected by centrifugation at 12,000 × *g* for 30 min at 4 °C and washed three times with 0.07% (v/v) 2-mercaptoethanol in acetone at minus 20 °C over night. The pellet was dried then suspended in 1 volume of 10 mm Tris-HCl pH 7.6.

Proteins from the culture filtrate were precipitated with 5 volumes of 10% (w/v) trichloroacetic acid, 0.07% (v/v) 2-mercaptoethanol in acetone at minus 20 °C for 4 h followed by washing as for mycelial proteins.

##### Sample Processing for LC-MS Analysis

Protein concentration of all samples was determined using the 2D-Quant kit (GE Healthcare, Little Chalfont, United Kingdom) according to the manufacturer's 'Standard procedure' protocol. Sample aliquots of 30–50 μg protein were reduced and alkylated as follows: Samples were diluted with 20 μl of 10% (w/v) sodium dodecyl sulfate (SDS), 10 μl of 1 m triethylammonium bicarbonate buffer (TEAB, pH 8.5) and the volume adjusted to 100 μl with milliQ water. Proteins were reduced with 1 μl of 1 m dithiothreitol (10 mm final conc.) overnight at 4 °C and 2 h at 22 °C and then alkylated with 5 μl of 1 m iodoacetamide (48 mm final conc.) in the dark at 22 °C for 2 h.

All mycelium samples treated with the Mem-PER Plus Membrane Protein Extraction Kit (Thermo Scientific) were cleaned up after reduction and alkylation using the 2D-Clean up kit (GE Healthcare) according to the manufacturer's Procedure A protocol. Removing Triton X introduced by the Mem-PER Plus Kit is crucial to prevent interference with downstream LC-MS analysis. Protein pellets resulting from the clean-up step were dissolved by adding 20 μl of 10% (w/v) SDS, followed by dilution to 100 μl with TEAB to 100 mm.

Sample proteins were co-precipitated with 0.5 μg of modified trypsin (Roche, Basel, Switzerland, sequencing grade) by adding 10 volumes of methanol as follows: One microliter of trypsin was added to the side of the Eppendorf tube and quickly flushed into the sample solution with 1 ml of 100% methanol at −20 °C. Tubes were incubated overnight at −20 °C. Protein precipitates were harvested by centrifugation at 20,000 × *g* for 15 min (4 °C). Pellets were washed twice, once with 1 ml of 90% (v/v) methanol at −20 °C and finally with 1 ml of 100% methanol at −20 °C and centrifuged each time as before. Protein pellets were briefly dried under a gentle stream of nitrogen. The final pellet was resuspended in 50 mm TEAB buffer (pH 8.5) containing 5% acetonitrile to obtain a 1 μg/μl peptide concentration by repeated vortexing and incubating the tubes for 1 min in the sonication bath. Samples were incubated at 37 °C for 2 h followed by the addition of further 0.5 μg of modified trypsin and 6 h digestion at 37 °C. Digests were stored at −80 °C until analysis.

##### LC-MS Analysis

For LC-MS/MS analysis, trypsin-digested samples were centrifuged for 5 min at 20,000 × *g* and an aliquot of 5 μl (peptide concentration approx. 1 μg/μl) was diluted to a volume of 20 μl with 6.5% formic acid prior to injection.

Tryptic peptides were analyzed on a Prominence nano HPLC system (Shimadzu, Kyoto, Japan) coupled to an LTQ-Velos Orbitrap ETD mass spectrometer (Thermo Fisher Scientific, Bremen, Germany). Mobile phases for chromatographic peptide separation were as follows: Eluent A was milliQ water containing 0.1% formic acid and eluent B was 80% acetonitrile/20% milliQ water (v/v) containing 0.1% formic acid. Sample aliquots of 20 μl (approx. 5 μg) were loaded onto a reversed-phase trap column (ReproSil-Pur C18-AQ 3 μm, 0.3 mm x 10 mm; Dr. Maisch, Ammerbuch-Entringen, Germany) and washed for 3.5 min at 30 μl/min using 100% eluent A. Peptide mixtures were subsequently back flushed onto a capillary column (150 μm x 150 mm) packed in-house with reversed-phase beads (ReproSil 100 C18 3 μm; Dr. Maisch) and separated applying two different gradients at a flow rate of 1 μl/min and 55 °C. A 90 min-gradient for samples PDC5 A, B, C, D: 0 min (7% eluent B) - 3.5 min (7% B) - 93.5 min (32% B) - 108 min (95% B) - 118 min (95% B) - 120 min (7% B) - 135 min (7% B); and a 180 min-gradient for samples PDC5E, F, G and PDC6B, C, E, F: 0 min (7% eluent B) - 3.5 min (7% B) - 170 min (35% B) - 180 min (45% B) - 190 min (95% B) - 200 min (95% B) - 202 min (7% B) - 218 min (7% B).

Column-separated peptides were electrosprayed into the mass spectrometer through a Nanospray Flex Ion Source (Thermo Fisher Scientific) using 30 μm inner diameter uncoated silica emitter (New Objective). Spray voltage was 1.5 kV with no sheath, sweep or auxiliary gases used. The heated capillary temperature was set to 250 °C and the S-lens to 50%.

The mass spectrometer was controlled using Xcalibur 2.2 software (Thermo Fisher Scientific) and was operated in positive ion and data-dependent acquisition mode to automatically switch between Orbitrap-full scan MS and ion trap- MS/MS acquisition. Full scan MS spectra (*m/z* 380 - 1700) were acquired in the Orbitrap mass analyzer with a resolving power set to 30,000 (at 400 *m/z*) after accumulation to a target value of 1 × 10^6^ in the linear ion trap. The top 20 most intense ions with charge states ≥ +2 were sequentially isolated with a target value of 5,000 and fragmented using collision-induced dissociation (CID) in the linear ion trap. Fragmentation conditions were set as follows: 35% normalized collision energy; activation q of 0.25; 10 ms activation time; ion selection threshold 1000 counts. Maximum ion injection times were 200 ms for survey full scans and 50 ms for MS/MS scans. Dynamic exclusion was set to 70 s and 90 s for 90 min- and 180 min-gradient runs, respectively. Lock mass of *m*/*z* 445.12 was applied with an abundance set at 0%.

##### Mapping Spectra to the Rhizoctonia and Wheat Genomes

Database searching; All MS/MS data were analyzed using Mascot (Matrix Science, London, UK; version 2.4.1) and SequestHT (Thermo Fisher Scientific, San Jose, CA; version 1.4.1.14). Databases used for both Mascot and SequestHT searches were the annotated *R. solani* AG8–1 genome [2] (13952 entries) and the 6-frame translation (1,729,543 entries) [12] or the wheat genome (ftp.ensemblgenomes.org/pub/plants/release-25/fasta/triticum_aestivum/dna/), supplemented in all cases with the contaminants database (247 entries, downloaded from maxquant.org on August 26, 2013). Mascot and SequestHT were searched assuming trypsin digestion with 2 missed cleavages, a fragment ion mass tolerance of 0.80 Da and a parent ion tolerance of 20 PPM. Carbamidomethyl of cysteine was specified in Mascot and SequestHT as a fixed modification. Deamidated of asparagine and glutamine and oxidation of methionine were specified in Mascot and SequestHT as variable modifications.

##### Experimental Design and Statistical Rationale

Five biological independent replicates for each sample type were collected. For efficient analysis the replicate samples were pooled for protein extraction and identification. Criteria for protein identification; Scaffold (version Scaffold_4.1.1, Proteome Software Inc., Portland, OR) was used to validate MS/MS based peptide and protein identifications. Peptide identifications were accepted if they could be established at greater than 95.0% probability by the Peptide Prophet algorithm (24). Protein identifications were accepted if they could be established at greater than 99.0% probability to achieve an FDR less than 1.0% and contained at least two identified peptides. Protein probabilities were assigned by the Protein Prophet algorithm (25). Proteins that contained similar peptides and could not be differentiated based on MS/MS analysis alone were grouped to satisfy the principles of parsimony. The mass spectrometry proteomics data have been deposited to the ProteomeXchange Consortium (26) via the PRIDE partner repository with the data set identifier PXD002806 and 10.6019/PXD002806.

##### Bioinformatic Analyses

Redundancy between the annotated proteins and proteins identified from the six frame translation of the genome was identified using BLASTclust (27). Any six frame proteins that shared greater than 95% identity over 95% of a protein from the annotated gene models were removed and a combined non-redundant protein list was created. Overlap between the proteins identified in each sample type was analyzed by using the programs venny (28) and venture (29). The entire set of annotated proteins from the *R. solani* AG8 genome (2) was supplemented with the nonredundant set of new proteins from the six frame translation of the genome having peptide support and gene ontologies were assigned using BLAST2go (www.blast2go.com). Enrichment analysis of sub-sets within the proteomic data compared with the entire genome, based on Fishers exact test, was also conducted using the BLAST2go program. Interproscan was used to further annotate functions onto proteins and categorisation of the ontologies was performed using AgBase GOSlim viewer (30). Secreted proteins were assigned to carbohydrate active enzyme (CAZy) categories using the CAZYmes Analysis Toolkit (31, 32). Motifs statistically over-represented in subsets were identified using MEME (33) using the following parameters -minsites 0 -nmotifs 25 -minw 4 -maxw 10 -p 8 -mod zoops.

##### Sequence Based Prediction of Secreted Proteins

SignalP version 3.0 using the D score algorithm and eukaryotic and 70 trimming options was used for prediction of likelihood of identified proteins being secreted using conventional secretory pathways. Although no well characterized secreted proteins from *R. solani* are available to establish appropriate conditions for prediction of secreted proteins for this species, SignalP 3.0 D score was found to be the most sensitive and accurate prediction tool for fungal secreted proteins (34).

##### Quantitative Polymerase Chain Reaction for Assessment of Gene Expression During Infection of Plant Hosts

Three replicate experiments (biological replicates) were conducted under the following conditions, each sample consisted of 4 pots containing 4 plants each. Four *R. solani* infected millet seed were added to each pot containing moist vermiculite and kept under humid conditions at 24°C for 1 week. Wheat or *Medicago truncatula* seeds were sterilized and germinated on moist filter paper in the dark at 4°C for 3 days. Seedlings were planted to pre-infected vermiculite and incubated at 16°C with 150 μm.m^−1^·s-^1^ light for 16 h per day. At 2 and 7 days after infection, seedlings were harvested and root tissue collected for RNA extraction using trizol reagent (Sigma). cDNA was produced according to (35) and quantitative PCR was performed using ssofast evagreen supermix (BioRad Berkeley, California) with primers listed in supplemental Table S1. Relative expression was calculated according to Anderson *et al.* (36). Statistical analyses were performed using the JMP 7.0 (SAS Institute, Cary, NC) software package.

##### Functional Assessment of Proteins for a Role in Fungal Pathogenicity

The open reading frames of candidate effector proteins were amplified from cDNA and cloned into the pK7WG2D plasmid (https://gateway.psb.ugent.be/vector/show/pK7WG2D)(37). Constructs containing the candidate effectors and the P19 silencing suppressor were co-infiltrated into *N. benthamiana* leaves according to (38, 39) and infiltrated regions of leaves inoculated with *R. solani* 3 days after infiltration. Lesions were measured and photos taken 5 days after inoculation with the pathogen. The NLP1 gene of *Fusarium oxysporum* 5176 was used as a positive control for induction of plant cell death.

## RESULTS

### 

#### 

##### Establishment of Infection Conditions

*R. solani* employs a necrotrophic lifestyle, killing plant cells to obtain nutrients and this plant cell death has been observed to precede the invading fungal hyphae, suggesting mobile pathogenicity factors are released from the fungus. The close association between the plant and fungus during infection meant it was not possible to obtain pure samples of fungal tissue for proteomic analysis of *in-planta* infection processes. To facilitate the collection of fungal samples with minimal plant protein contamination, an infection system was developed that enabled collection of fungal mycelium, culture filtrate and infected plant host samples with negligible observed cross contamination of tissue and no protein spectra showing a greater likelihood of a host rather than fungal origin. QPCR analysis of *R. solani* genes previously found to be up-regulated in infected wheat roots from a soil inoculation system compared with vegetative grown mycelium (2) was performed on infected wheat roots from the infection system used in this study. Of the 29 genes tested, the majority showed strong up-regulation in the infected wheat roots from the Petri dish infection system (Table I).Expression of the wheat ADP-ribosylation factor gene could be detected in the wheat root samples but not in the distant hyphae samples from the Petri dish infection system (data not shown). The results suggest that a reliable infection was occurring under these conditions and the distant hyphae could be collected without measurable contamination with wheat tissue.

**Table I:** Gene expression values for selected infection up-regulated R. solani genes under infection conditions

	Vegetative^a^		Wheat roots; Petri dish infection^b^		Wheat roots; soil infection^c^	
	Mean	Standard error	Mean	Standard error	Mean	Standard error
RSAG8G_00146	1	0.2	12.2	1.2	30.9	13.4
RSAG8G_00435	1	0.4	62.7	2.1	12.7	4.8
RSAG8G_02244	1	0.2	2.1	0.1	49.4	32.9
RSAG8G_02343	1	0.1	0.7	0.1	20.8	15.9
RSAG8G_02727	1	0.3	99.8	13.1	34.5	6.5
RSAG8G_03246	1	0.3	3.5	0.2	14	4.9
RSAG8G_03830	1	0.2	1	0.1	22.6	4.8
RSAG8G_03874	1	0.3	1.5	0.1	566.7	224.4
RSAG8G_04390	1	0.2	1.1	0.1	58.4	28.8
RSAG8G_04560	1	0.5	10.6	2.9	49	22.4
RSAG8G_05829	1	0	0.6	0	14.6	5.8
RSAG8G_05942	1	0	181.8	5.7	98.7	29.7
RSAG8G_05942	1	0.3	106.3	5.2	98.7	29.7
RSAG8G_06853	1	0.1	0.4	0	27.2	12.5
RSAG8G_07318	1	0.1	0.6	0	45.2	27
RSAG8G_07380	1	0.2	1.1	0.1	26.4	9.4
RSAG8G_07433	1	0.4	43.5	1.8	9.9	4.7
RSAG8G_07489	1	0.2	322	29.6	285.2	217.2
RSAG8G_07504	1	0.2	4.7	0.1	120	85.8
RSAG8G_07875	1	0.3	13.1	0.4	20	6.4
RSAG8G_08039	1	0.4	11.8	2.5	29.4	5.9
RSAG8G_09107	1	0.1	5.1	0.4	19.6	4.9
RSAG8G_09852	1	0.7	0.1	0	853.1	122.7
RSAG8G_10274	1	0.1	2.8	0.4	37.1	16.2
RSAG8G_11016	1	0.1	295.9	20.6	251.7	97.7
RSAG8G_11570	1	0.2	0.2	0	11.7	4.5
RSAG8G_12186	1	0.2	4.3	0.3	27.6	1.2
RSAG8G_13658	1	0.7	0.6	0.2	426	131.2
RSAG8G_03280	1	0.7	140.8	3.8	4143.8	465.1
Control (Beta-catenin)	1	0	1.8	0	1	0.2
Control (C3HC4 zinc finger)	1	0	0.2	0	0.9	0.2
Control (Ribosomal protein L14)	1	0	0.9	0	1	0.2

^a^*R. solani* in minimal medium alone.

^b^Infected wheat roots from Petri dish infection conditions.

^c^Infected wheat roots from an in-soil infection.

##### Mapping of Spectra to R. solani AG8 Genome

Mapping of spectra from the LTQ Orbitrap Velos to the genome of the *R. solani* AG8 isolate WAC101035 identified a total of 1,350 unique proteins relating to previously annotated genes and 1310 proteins relating to a six-frame translation from all ATG codons in the genome (Table II, supplemental Table S2). Redundant protein sequences from the annotated and six-frame translation sets which shared a minimum of 95% identity across at least 95% of the annotated protein sequence according to BLASTp were conflated with the manually annotated proteins, producing a list of 2,086 unique proteins identified across all samples (Table II, supplemental Table S2).

**Table II:** The number of mapped R. solani peptides, identified proteins, and estimation of the peptide FDR

	Protein location	Condition	Time point	Annotated proteins			Six frame translation of genome sequence		
				Mapped peptides	Identified proteins	Protein FDR	Mapped peptides	Identified proteins	Protein FDR
PDC5A	Soluble mycelium	Vegetative growth	3 dpi	2977	260	0.4%	1437	170	0.0%
PDC5B	Soluble mycelium	Vegetative growth	7 dpi	3471	317	0.3%	1712	218	0.0%
PDC5C	Soluble mycelium	Infection	3 dpi	1539	150	0.7%	1023	103	0.0%
PDC5D	Soluble mycelium	Infection	7 dpi	1682	199	0.0%	1013	131	0.0%
PDC6B	culture filtrate	Vegetative growth	7 dpi	6008	143	0.0%	2950	79	0.0%
PDC6C	culture filtrate	Infection	3 dpi	2692	69	0.0%	1026	47	0.0%
PDC5E	culture filtrate	Infection	7 dpi	2059	113	0.9%	1379	61	0.0%
PDC5F	Membrane	Vegetative growth	3 dpi	8020	751	0.3%	4154	574	0.0%
PDC6E	Membrane	Vegetative growth	7 dpi	10273	869	0.1%	5318	832	0.0%
PDC5G	Membrane	Infection	3 dpi	7492	676	0.0%	3943	532	0.0%
PDC6F	Membrane	Infection	7 dpi	7467	637	0.0%	4144	559	0.0%
All samples				60630	1350	1.00%	31138	1310	0.00%
Non-redundant proteins after clustering							2086		

To assess whether any spectra may relate to wheat proteins within the samples, spectra were also submitted against the wheat genome database. Overall no proteins were found to have a better hit in the wheat database than in the *R solani* database. Moreover, the only proteins with potential hits to both the *R. solani* and wheat databases were found in both *R. solani* vegetative (no wheat present) and infection samples (Supplemental table S3) suggesting they are of fungal origin. No proteins that were only found in infection samples had spectra matches to wheat.

**Fig. 1.**
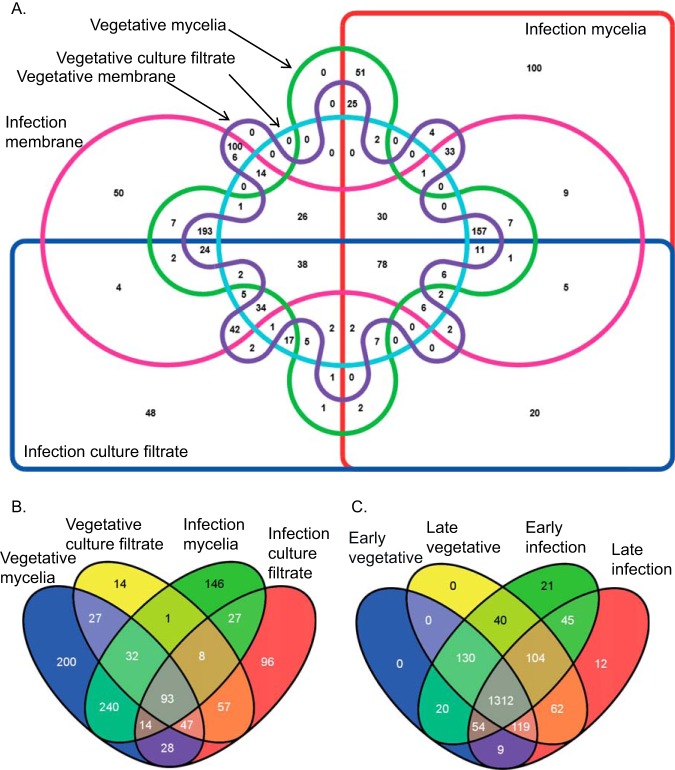
**Venn diagrams representing the overlap among protein sample types.** *A*, All sample types with time points combined. *B*, Mycelia and culture filtrate from vegetative and infection conditions. *C*, early and late time points for vegetative and infection conditions.

Analysis of the proteins found in each sample type indicated that there was a relatively low proportion of proteins identified in more than one sample location (soluble proteins from mycelia, membrane bound proteins from mycelia or the culture filtrate) suggesting that the sampling isolated largely different pools of proteins (Fig. 1*A*). A greater number of proteins were identified from membrane samples compared with soluble mycelia and culture filtrate samples (Table II). A total of 1006 proteins were identified in both vegetative and infection membrane samples but not in other sample types, an additional 50 were specific to infection membrane samples and none were only found in vegetative membrane samples (Fig. 1*A*, supplemental Table S4).

To gain a clearer perspective on the proteins identified from mycelia and culture filtrate samples, Venn diagrams were produced using data only from these samples types (Fig. 1*B*). Relatively few proteins (14) were identified only in the culture filtrate under vegetative conditions and these were also found in membrane samples. By contrast, 96 proteins were found only in infection culture filtrate, indicating more proteins may be produced and secreted from the fungus under these infection conditions. Twenty-one percent (27 proteins) of all proteins identified in culture filtrate under infection conditions were also identified in mycelia from infection conditions, suggesting the separation of culture filtrate and mycelia sample types was reasonably effective. Approximately one third of the proteins identified in mycelia samples were found in both vegetative and infection samples with approximately equal distribution of the remaining two thirds of proteins across vegetative and infection samples suggesting relative large differences in protein accumulation were observed under the two conditions.

Characterizing the likelihood of the identified proteins to be secreted using SignalP 3.0 (40, 41) revealed 21.2% of culture filtrate proteins, 11.3% of mycelial proteins and 5.1% of membrane proteins were predicted to be secreted.

Comparisons were also conducted by grouping samples based on the time point at which they were taken (Fig. 1*C*). A total of 78 proteins were found only in infection conditions with 21 proteins specific to the early time point and 12 specific to the late time point, suggesting some changes in biological activities may be occurring over time. Early vegetative samples had more in common with early infection than with late infection samples. Late vegetative had more in common with late infection samples than early infection suggesting an underlying adaptation, perhaps to lower nutrient availability, is occurring over the time course. All proteins identified in vegetative conditions were also identified in infection conditions, suggesting that overall, no protein degradation or down-regulation of vegetative protein synthesis was observed. However, changes in protein localization between vegetative and infection samples may be occurring because analysis of locations separately (Fig. 1*B*) does show proteins identified only in vegetative samples.

To further investigate the types of proteins identified in the different samples, the BLAST2GO and GOSlim programs were used to study the predicted cellular location of the proteins. A large component of the proteins from the mycelia samples were classified as cellular component, intracellular, cell and cytoplasmic (supplemental Fig. S2). Proteins from the culture filtrate also had a large component classified as “cellular component,” but increases in the number of proteins classified as extracellular region, endoplasmic reticulum and golgi apparatus, which are locations associated with conventional protein secretion. Membrane proteins had slightly increased organelle and cytoplasmic membrane bound vesicle categories and a reduced cytoplasm category (supplemental Fig. S2).

##### Assessment of Putative Protein Function

**Fig. 2.**
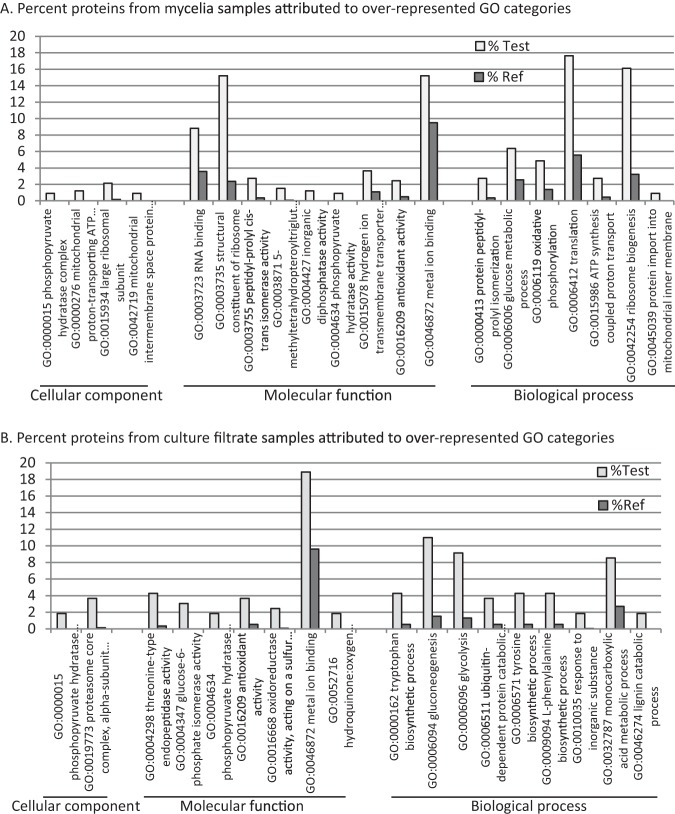
**GO categories over-represented in sample types according to Fishers' exact test, FDR 0.05.** Histograms present the percentage of proteins in a sample having a GO term annotation belonging to each GO category. % Test is the percent of proteins in samples (mycelia or culture filtrate) attributed to each group. % Ref is the percent of proteins in a random selection of proteins from the *R. solani* AG8 genome attributed to each group.

Over-represented GO terms relating to proteins in each sample type relative to a random selection of proteins from the genome sequence was analyzed using Fishers exact test. Statistically over-represented GO categories for the mycelia and culture filtrate sample types are presented in Fig. 2. Categories over-represented in the mycelial sample types mainly reflected aspects of primary metabolism (Fig. 2*A*). Categories over-represented in the culture filtrate samples included catabolism of lignin, a major component of plant cell walls (Fig. 2*B*). Function, process and cellular location terms associated with proteosome mediated protein degradation were found to be over represented in the culture filtrate as were three function terms associated with management of the redox state. Other categories included response to inorganic substance and biosynthesis of amino acids.

As the number of proteins identified from membrane samples was higher than for mycelium and culture filtrate, there were many more statistically over-represented GO categories for these sample types (supplemental Table S5). Over-represented cellular locations included the clathrin coat of golgi associated vesicles that interface with endosomal transport, a process shown to be required for virulence of smut fungi on their plant hosts (65), ATP synthase complex and mitochondrial inter-membrane space. Many of the remaining molecular functions related to ion binding or transport and a wide variety of metabolism processes.

##### Proteins Associated with Infection Conditions and the Culture Filtrate

**Fig. 3.**
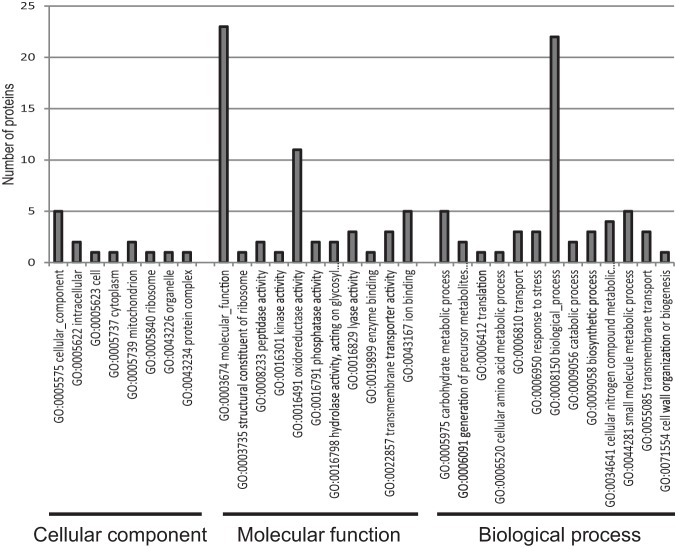
**Representation of GO terms according to GOSlim for proteins unique to infection conditions.** The number of proteins per category is presented.

Of particular interest for this destructive pathogen of many crop species are the proteins potentially associated with causing disease on crop hosts. As mentioned previously, no proteins found exclusively in infection samples had spectra hits to the wheat database, strongly suggesting they were of *R. solani* origin. Functional classification of proteins only identified in infection conditions using Interproscan and GOSlim found a high proportion were putative oxidoreductases, peptidases or belonged to plant cell wall degrading enzyme categories such as glycosyl hydrolase, various types of lyase, carbohydrate metabolic processes, and cell wall organization or biogenesis (Fig. 3, supplemental Table S6). Several other categories, such as response to stress and membrane transport may also be associated with pathogenesis functions.

**Fig. 4.**
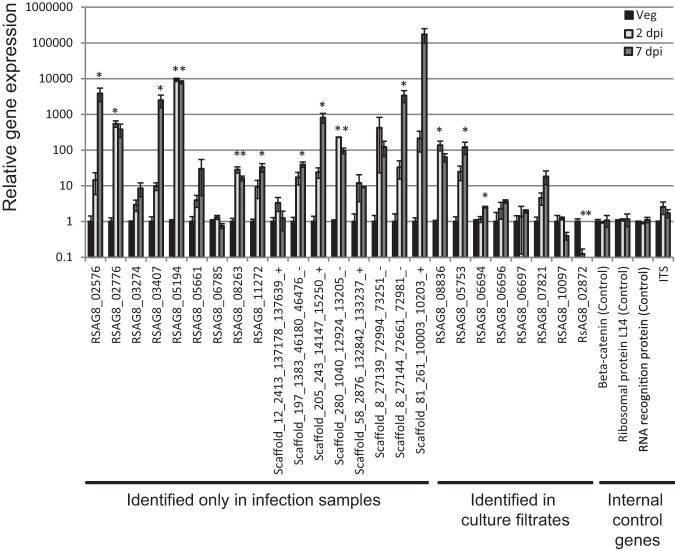
**Expression levels for selected proteins identified only in infection samples in wheat roots infected with *R. solani* AG8 and vegetatively grown mycelium.** Solid bars represent expression in vegetative conditions, light gray bars are infected wheat roots harvested 2 days after inoculation, dark gray bars are infected wheat roots harvested 7 days after inoculation. Asterisks indicate a significant difference to the expression observed under vegetative conditions according to Dunnetts' test, *p* < 0.05.

To confirm if the proteins identified only in infection conditions were up-regulated during an in-soil infection we selected 17 genes for quantitative PCR analysis. The expression of these genes was determined in vegetative mycelium and mock treated or infected wheat roots at 2 and 7 days after inoculation. Six out of the nine genes previously annotated in the genome showed significant up-regulation in wheat root tissue infected with *R. solani* AG8 when compared with vegetative mycelium according to Dunnetts' test (*p* < 0.05). Four out of eight proteins identified from the six frame translations of the *R. solani* AG8 genome had transcripts up-regulated *in-planta* (Fig. 4). These findings support the pathogenesis related expression of the proteins only identified in the infection conditions and the identification of previously uncharacterized genes from the six frame translation of the genome. Some of the other infection only proteins may be regulated post-transcriptionally or their transcription induction may occur at different time points.

To further characterize plant cell wall degrading enzymes present in the culture filtrate samples, proteins were compared with the CAZy database for carbohydrate active enzymes (Supplemental table S7). Consistent with the important role of these enzyme classes in the infection process of *R. solani* (42), a wide variety of CAZy categories (44 different groups) were represented. Prevalent among these groups were enzymes with predicted activity against plant cell wall components including cellulose. The most highly represented categories included CMB13 (carbohydrate-binding), CE4 (deacetylase/esterase activity), and GH16 (glycoside hydrolase). Consistent with the lower amount of pectin in the cell wall of wheat and other monocots, only one pectate lyase enzyme was identified in the culture filtrates (supplemental Table S7) despite the *R. solani* AG8 genome containing 14 PL3 and 22 PL1 members. Interestingly, a large proportion of the members of CBM13 (13 out of 15 proteins) were directly classified by pfam or had highest homology to proteins classified by pfam as containing ricin-like lectin domains. Although these proteins appear to possess the ricin-B carbohydrate binding domain, they appear to be devoid of the catalytic ricin-A domain that confers function to the plant toxin, ricin.

**Fig. 5.**
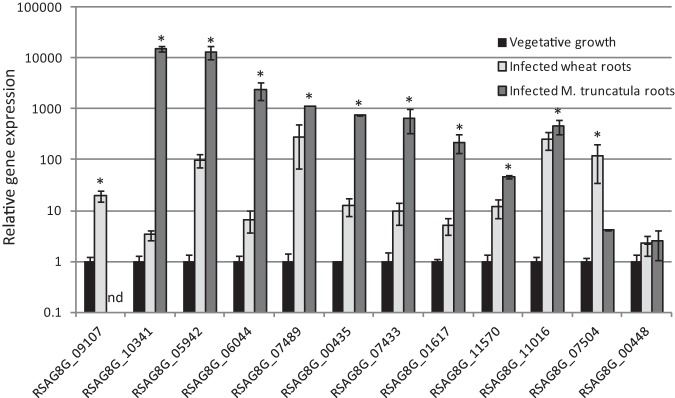
**Expression levels for *R. solani* AG8 genes predicted to encode pectate lyases during vegetative growth, infection of wheat roots and infection of *Medicago truncatula* roots.** Solid bars represent expression in vegetative conditions, light gray bars are infected wheat roots harvested 7 days after inoculation, dark gray bars are infected *M. truncatula* roots harvested 7 days after inoculation. nd, not detected. Asterisks indicate a significant difference to the expression observed under vegetative conditions according to Dunnetts' test, *p* < 0.05.

To further investigate if the expression of cell wall degrading enzymes is tailored to the host plant being infected, the expression of randomly selected members of the pectate lyase and pectin esterase families of *R. solani* AG8 during infection of wheat and the legume, *Medicago truncatula* was determined using quantitative PCR. Nine of the 12 selected genes were significantly up-regulated during infection of medicago whereas up-regulation during infection of wheat was greatly reduced (Fig. 5) with only one gene being significantly up-regulated. These findings indicate the pathogenesis mechanisms are tailored to the particular host being infected such as a preference for up-regulation of pectate degrading enzymes when infecting a host with high levels of pectin in the cell walls.

##### Analysis of Over-represented Motifs

Previously analysis of proteins secreted from fungal plant pathogens has identified conserved motifs that are critical for the function of the protein in pathogenesis (43). To identify any conserved motifs present in the proteins identified in this study, MEME was used to search proteins from the culture filtrate of infection conditions and particular attention was paid to motifs with a conserved position within the proteins as a further indicator of conserved function.

One of the motifs with the highest significance was [LysGln][SerThr]Phe[AspAsn]Trp[ThrAla][Ser.Glu.]PheLysAsn whichwas identified in 15 proteins. In 11 of these proteins the domain started between residue positions 31 and 47 and two others at 67 and 71. Two other proteins possessed the motif further toward the C terminus at residues 134 and 425. The majority of proteins which contain the motif were classified in an interproscan search as potential delta endotoxins. Consistent with the identification of these proteins in both infection and vegetative samples, the expression of four delta endotoxin genes (RSAG8_06694, RSAG8_06696, RSAG8_06697and RSAG8_07821) were found to be relatively consistent between vegetative mycelium and infected root tissue, whereas RSAG8_05753 was significantly up-regulated at 7 days after inoculation (Fig. 4).

Another over-represented motif [TyrArg]AsnAlaCysProPheThrlleTrpPro was found in five proteins beginning at positions 22–70 residues from the N terminus. Proteins containing this motif were classified as thaumatins, previously considered to be plant anti-microbial proteins. A third over-represented motif identified was [TyrPhe]Tyr[Ser.Asp.]TyrPheLys[AsnLysGln][AlaGlyArg]CysPro which was found toward the C-terminal end of four of the five putative thaumatins, plus an additional protein. The expression of one thaumatin gene (RsAG8_08836) was found to be significantly up-regulated in infected root tissue at 2 days post-infection (Fig. 4).

##### Assessment of Protein Function During Infection

**Fig. 6.**
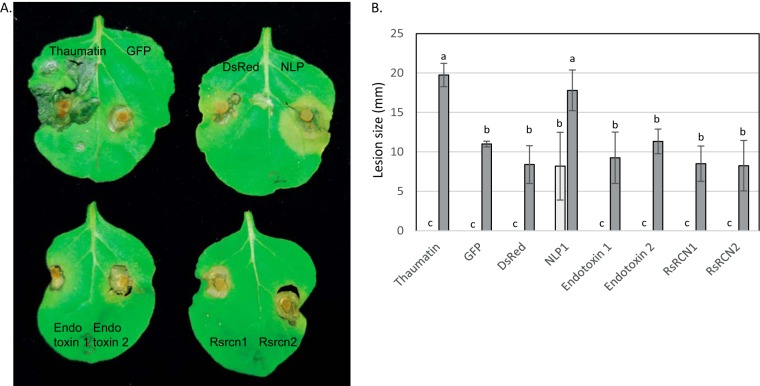
**Functional tests of proteins for a role in promoting susceptibility to *R. solani*.** *A*, Photos of agroinfiltrated *N. benthamiana* leaves expressing recombinant proteins and subsequently inoculated with *R. solani. B*, Lesion sizes on *R. solani* inoculated *N. benthamiana* leaves expressing recombinant proteins, dark gray bars. Light gray bars, necrotic lesion on agroinfiltrated leaves without *R. solani* inoculation. The average and standard error of four replicates are shown. Columns not connected by same letter are significantly different according to Student's *t* test. With the exception of NLP1, all leaves not inoculated with *R. solani* has a lesion size of 0. The experiment was repeated three times with similar results. Thaumatin, RsAG8_08836; GFP and DsRed, fluorescent reporter genes used as controls; NLP1, Necrosis and ethylene inducing protein like protein from *F. oxysporum* 5176 (FOXB_01740); Endotoxin 1, RSAG8G_05753; Endotoxin 2, RSAG8G_06697; RsRCN1, RSAG8G_10097; RsRCN2, RsAG8_02872.

A total of 15 proteins were chosen for assessment for a role in pathogenicity based on their identification in infection conditions, gene expression during infection and/or the putative functions assigned by interproscan. As no genetic transformation system for gene knock out is presently available for *R. solani*, we introduced the open reading frames of candidate effectors under the control of the CaMV 35s promoter into *Nicotiana benthamiana* using agroinfiltration and lesion size from subsequent infection with *R. solani* was measured. Several *R. solani* isolates are known pathogens of tobacco, causing leaf spot and root rot and inoculation of *N. benthamiana* with *R. solani* in the present study caused the development of water soaked lesions similar to those previously described (44). All expression vectors also contained a cassette for constitutive expression of GFP which was used to confirm transformation and transgene expression had occurred prior to *R. solani* inoculation (data not shown). Expression of RSAG8_08836, a thaumatin protein, increased the size of lesions formed following *R. solani* inoculation in comparison to control areas expressing *GFP* only or *GFP* and *DsRed* (Fig. 6*A*, 6*B*). Positive control infiltrations with the *NLP1* gene from *Fusarium oxysporum* (Fo5176) which is known to induce plant cell death, showed similar increased lesion size (Fig. 6). All other infiltrated proteins did not alter lesion size (Fig. 6 and data not shown).

## DISCUSSION

The necrotrophic fungal pathogen *Rhizoctonia solani* imposes considerable losses to a wide range of crops worldwide including wheat, rice, barley, maize, soybean, potato, sugarbeet, and many others (1). The AG8 isolate WAC10335 and causes significant losses to wheat, barley, canola, and various legume crops (21, 23). The recently published genome sequence of AG8 (2) and other *R. solani* isolates (20, 45–47) provide an excellent basis for improving our understanding of the metabolic and pathogenic processes of this intractable pathogen.

Besides producing a litany of cell wall degrading enzymes, very little is known about the pathogenicity mechanisms of *R. solani*. A rice-infecting isolate from AG1-IA has been shown to produce several host specific metabolite toxins consisting of phenyl acetic acid or its derivatives, a phenolic compound or a carbohydrate (48–51) and corresponding quantitative susceptibility loci have been identified in some rice varieties (52, 53). However, despite genome sequences with auto- or manually annotated gene models, little is known about the proteins produced by *R. solani* under vegetative growth or infection of a plant host. In some systems, growth of the pathogen in specific minimal medium is sufficient to induce the production of toxins/effectors and their secretion into the culture filtrate (54, 55). To obtain proteomics data for as many expressed genes as possible, samples were collected from vegetative growth in minimal medium as well as infection of wheat. Proteins were also purified from various cellular locations including membrane-bound proteins from mycelia, intracellular soluble proteins from mycelia and culture filtrates. Comparison of the expression of genes previously associated with pathogenesis by Hane *et al.* (2) in infected wheat roots and the infection system used here, revealed that 62% of genes were up-regulated under both conditions (Table I). Unlike infection from simultaneous germination of spores applied to a leaf, infection of *R. solani* from mycelia does not provide synchronized infection of inoculated plant tissue. Rather, different parts of the plant are exposed to initial infection attempts at different times and this may be related to some of the observed differences in gene expression between the soil infection and Petri dish infection systems used here. Although some pathogenesis-related proteins may not be found by studying the pure hyphae, distant to the infected roots, these findings support the potential of the system to identify *R. solani* proteins up-regulated during infection of a plant host while minimizing the potential for contamination of fungal protein samples with the far more abundant host proteins, particularly at the early stages of infection.

The relatively low proportion of proteins identified in multiple different sample types; membrane, intracellular soluble proteins and culture filtrate, suggest the sampling technique provided reasonably reliable separation and that *R. solani* AG8 expresses quite distinct sets of proteins in each of these locations (Fig. 2*A*). Although all of the proteins identified in the vegetative growth conditions were also observed during infection, an additional 78 *R. solani* proteins were only observed during infection suggesting an adaption to the presence of the plant host was occurring. Mapping of spectra to the wheat genome database did not identify wheat as a potential source of these infection only proteins.

Although the overall percent of proteins identified in culture filtrate samples predicted to be secreted by *in-silico* methods was reasonably low (21.2%) it was much larger for this sample type than intracellular or membrane samples, supporting the enrichment for secreted proteins in culture filtrate samples. Neighboring genes in fungal genomes have a high proportion of over-lapping transcripts (56) and this may lead to impaired accuracy in predicted secreted proteins by conventional methods. Nonetheless, the percentage of proteins predicted to be secreted in-silico is in line with previous studies of fungal culture filtrates. For example, Meijer *et al.* (57) found 28% of proteins from the culture filtrate of *Phytophthora infestans*, the potato late blight pathogen, to be predicted to be secreted. The SignalP 3.0 D score algorithm was shown to be one of the most sensitive and accurate methods to predict fungal protein secretion (34), a low proportion of culture filtrate proteins predicted to be secreted by SignalP 3.0 may have been influenced by several factors. One of these may be a substantial degree of non-conventional secretion of leaderless extracellular proteins occurring in the fungus. Several studies suggest that non-conventional secretion pathways including exocytosis/golgi-independent secretion, autophagosomes, and (MVB-mediated) extracellular vesicle secretion may be responsible for translocation of a large proportion of extracellular proteins, some having important functions in hyphal tip growth and pathogenesis (58–62). The details of these pathways are largely unknown and without the ability to predict which proteins may be exported through these pathways, proteomics studies to identify extracellular proteins remain an important method to understand fungal pathogenesis (57).

To further study the types of proteins identified in the culture filtrate and other samples, putative functions were assigned based on sequence similarity and proteins were subsequently attributed to gene ontology (GO) terms. As might be expected, membrane proteins identified were rich in functions/processes related to transport, secretion and signaling. In particular, an over-representation of proteins associated with the clathrin coat of golgi associated vesicles was observed. These vesicles are associated with endocytosis from the plasma membrane and endosomal trafficking to the golgi and various locations within the cell, including the nucleus (63) (64). Bielska *et al.* (65) report an essential role for long range endosomal signaling during the pathogenicity of a fellow basidiomycete and plant pathogenic fungus, *Ustilago maydis*. Interference with the transport of early endosomes from the infecting hyphal tip to the distant nucleus reduced secretion of effectors into the host plant. Other studies have identified critical roles for membrane proteins in fungal pathogenesis and membrane functions such as endocytosis and autophagy as prerequisites for successful pathogen invasion, particularly at early stages (66–69).

As infections or vegetative growth matures, nutrient availability alters and requires adaptation of metabolism by the fungus. Analysis of proteins only identified at the late time point revealed five proteins associated with carbohydrate metabolic process suggesting that the fungus may be adapting to a reduced supply of complex carbon. A similar trend was observed in previous studies of sclerotia production in a rice infecting *R. solani* AG1-IA isolate (70). *R. solani* produces sclerotia as over-wintering structures to survive for long periods in the soil in the absence of a plant host. Typically, these highly melanized structures of matted hyphae are produced as nutrient resources become scarce (1). Kwon *et al.* (70) identified numerous proteins relating to carbohydrate metabolism changing in abundance during sclerotia production including the sustained up-regulation of a formate dehydrogenase. These proteins are proposed to function in the glyoxylate cycle which is activated in microbes when complex carbon sources are not available and have been linked to pathogenicity in some fungi (71).

In contrast to membrane and mycelia samples, no significant GO term enrichments could be detected for the proteins found only in the culture filtrate under infection conditions. This may in part relate to the small number of proteins in the set, however, another contributor is likely to be the low rate of GO term annotation. Within this protein set the majority of proteins were novel, having no similarity to previously characterized proteins. A high rate of novel proteins secreted from fungal pathogens of plants has previously been described, *e.g.* for proteomic analysis of isolated haustoria of *Blumeria graminis* f. sp *hordei* infecting barley (72) and for the predicted secretome of *Ustilago maydis* (73). Furthermore, Saunders *et al.* (74) suggest that fungal effectors with a role in pathogenesis are likely to be novel proteins with infection related expression patterns such as those identified in this set. Further investigation into the functions of the infection related extracellular proteins from *R. solani* is ongoing.

Effector proteins in other filamentous pathogens of plants have been found to possess conserved effector motifs associated with their function, for example that allow entry into plant cells (7, 75). The potential for new or previously identified motifs in *R. solani* was explored by searching for conserved motifs that are over-represented in the secreted proteins identified here. In addition to a conserved sequence, effector motifs also commonly possess a conserved start position in the proteins in which they are found (76, 77). Using these criteria for *R. solani* did not identify a general effector motif associated with secreted proteins, but did identify several motifs that are associated with particular protein families. In particular, two motifs associated with thaumatins were found in five proteins. Initially identified as plant defense proteins, thaumatins have recently been found in fungi, nematodes and a wide range of plants. Consistent with their wide distribution, a diverse array of functions has been ascribed to the family including protease inhibiting, glucan binding, xylanase inhibiting and signaling functions (reviewed in (78)). Another motif was found to be associated with delta endotoxins, commonly described as anti-insect defense proteins in bacteria including *Bacillus thuringiensis* (79). To date there has been no link to ecological fitness nor plant pathogenesis described for delta endotoxins from fungal origins.

One of the statistically over-represented GO categories in the culture filtrates of vegetative and infection samples was lignin catabolism which supports an enrichment of the fraction for proteins secreted from the fungus to modify plant cell walls. Further analysis of potential carbohydrate active enzymes (CAZy) in the culture filtrates found 44 CAZy categories to be represented. Although a wide diversity of categories was identified, only one protein (RSAG8_07489) was predicted to be involved in pectin degradation. On a genomic level, the pectate lyase family is expanded in the dicot and monocot infecting AG8 isolate of *R. solani* (14 PL3 and 22 PL1 members) relative to the rice infecting AG1-IA isolate (1 PL3, 7 PL1 members) whereas gene families related to catabolism of xylan, a major cross-linking component of monocot cell walls, did not differ significantly (2, 45). The low representation of pectin related activities may be associated with the use of wheat as the plant host in the infection conditions. Cereals and other monocot plants, have a low proportion of pectin in the cell wall compared with dicot plants (80). The preference for pectin degradation in dicot pathogens compared with monocot pathogens has been previously reported (81) but specific adaptation of the infection process in a broad host range pathogen (of monocots and dicots) has not been described to our knowledge. To further investigate this, the expression of a random selection of genes in the pectate lyase family in *R. solani* AG8 was determined during infection of wheat and the dicot *Medicago truncatula*. Nine of the 12 tested genes were significantly up-regulated during *M. truncatula* infection and only one significantly up-regulated during wheat infection. Together these findings suggest adaptation of the pathogenicity strategy to the individual host is occurring at both the gene expression level and at the genome level through expansion of the gene family.

Other functional categories over-represented in the culture filtrate samples included three related to the redox state, two oxidoreductase categories and one antioxidant activity category. The importance of plant produced reactive oxygen species (ROS) in resistance to numerous plant pathogenic fungi has been described by many studies (82). ROS production from the mitochondrial electron transport chain was demonstrated to be required for full plant resistance to *R. solani* AG8 (83) and a double loss-of-function mutation in plasma membrane located RBOHD and RBOHF was shown to render plants highly susceptible to the same AG8 isolate (22). Furthermore, proteomics analysis of the enhanced resistance of tobacco to *R. solani* infection following inoculation with the plant growth promoting fungus *Trichoderma harzianum* identified oxidative stress responses to be one of the main protein categories associated with the *T. harzianum* induced resistance to *R. solani* (84). The production of ROS has several functions in the plant response to microbe invasion, including potentiating cell death at the site of infection, preventing the uncontrolled spread of cell death from the infection site and regulating the levels of plant defense hormones jasmonic acid, ethylene and salicylic acid in nearby cells (85, 86). The response of redox managing proteins observed in this study may be associated with limiting the unfavourable direct effects of ROS on the pathogen or as a mechanism to manipulate ROS levels to drive fungal pathogenesis and development, as has been implicated in some necrotrophic fungal pathogens (87, 88). For example, another broad host range necrotizing fungal pathogen of plants, *Sclerotinia sclerotiorum*, was shown to produce oxalic acid to suppress an initial host ROS response and oxalic acid deficient mutants unable to manipulate early host ROS were less pathogenic (89). Analysis of *R. solani* proteins only identified under infection conditions also found ROS related functions, including oxidoreductases, as highly represented. Many other functional or process categories were associated with modification/degradation of the plant cell wall including glycosyl hydrolase, lyase and cell wall organization or biogenesis whereas other categories such as membrane transport and response to stress may also be associated with pathogenesis. Overall, 10 out of 17 proteins selected for confirmation of pathogenesis related expression in infected wheat roots showed significant up-regulation in comparison to vegetative mycelia at the two time points selected, supporting a role for these proteins in the infection process of *R. solani.* Other genes not found to be up-regulated may be post-transcriptionally regulated, regulated at other time points during infection or not have pathogenesis related expression. The proteins specifically identified in infection conditions provide good leads for further investigation of the pathogenesis mechanisms of this relatively uncharacterized but important fungus.

The observation of increased lesion size from *R. solani* following expression of a thaumatin like protein, RsAG8_08836, in *N. benthamiana* agroinfiltrations suggests this protein may have a role in pathogenicity of *R. solani* either through facilitating infection or manipulating/suppressing host defense responses. Follow-up studies involving the development of a genetic transformation system allowing gene knockout in *R. solani* would enable further functional testing of genes such as RsAG8_08836 which are putatively involved in pathogenesis. Thaumatin like proteins are a large family of proteins for which diverse functions have been attributed to individual members including glucan binding, xylanase inhibitor, protease inhibitor activities, the accumulation of reactive oxygen species and the activation of yeast apoptosis (90); (91). Initially discovered in plants as pathogenesis related defense proteins, members of the family have more recently been discovered in nematodes (92), insects (93) and fungi including *R. solani* (94, 95) were the protein, which has low similarity to the protein RsAG8_08836 identified in this study, exhibited strong B-1,3-glucanase activity and was able to lyse living *Saccharomyces cerevisiae* (94). Interestingly, the overexpression of a thaumatin family member from the plant *Thaumatococcus danielli* in tobacco resulted in enhanced resistance to *R. solani* (44, 96), suggesting a variety of functions are performed by members of this diverse family of proteins. The mechanism by which the expression of RsAG8_08836 in plants increased lesion sizes is unknown. The potential for a role for RsAG8_08836 in the manipulation of host cell death and reactive oxygen species accumulation in response to *R. solani* warrants further investigation as a lead to understanding the mechanisms this recalcitrant pathogen uses to cause disease in its broad range of host plants.

## CONCLUSIONS

Proteomics analysis of *R. solani* AG8 under vegetative and infection conditions has been a productive approach to identifying a substantial number of proteins in support of previous gene models, and potential new proteins from six frame translation of the genome. Many of the proteins found in the culture filtrate had predicted functions relating to modification of the plant cell wall, a major activity required for pathogenesis on the plant host. Other secreted proteins have provided leads for further investigation into the specific mechanisms of pathogenesis employed by *R. solani* AG8. The expansion of the pectate lyase family in the AG8 genome and the coordinated regulation of pectate metabolism enzymes depending on the cell wall components of the host being encountered has provided insight into how a single fungal isolate is able to induce disease on a very broad range of host plants. The utility of this approach for uncovering the molecular mechanisms of pathogenesis for uncharacterized pathogens is also demonstrated by the increased susceptibility of plants expressing a *R. solani* thaumatin gene. It is anticipated a diverse array of pathogenesis mechanisms are employed to infect the broad range of host plants and further investigation of this is necessary for the design of robust resistance strategies effective across several crops impacted by *R. solani* diseases.

## Supplementary Material

Supplemental Data

## Footnotes

^1^ The abbreviations used are:

NLP: necrosis and ethylene inducing protein-like proteins
