# Supplementary material for: Proteomic Analysis of Rhizoctonia solani Identifies Infection-specific, Redox Associated Proteins and Insight into Adaptation to Different Plant Hosts
Source: Mol Cell Proteomics. 2016 Jan 25;15(4):1188–203. doi: 10.1074/mcp.M115.054502 (PMC4824849; doi:10.1074/mcp.M115.054502)
Supplement: Supplemental Data [file supp_15_4_1188__index.html]

Proteomic analysis of Rhizoctonia solani identifies infection-specific, redox associated proteins and insight into adaptation to different plant hosts — Proteomic Analysis of Rhizoctonia solani Identifies Infection-specific, Redox Associated Proteins and Insight into Adaptation to Different Plant Hosts — Proteomic Analysis of Rhizoctonia solani — Supplemental Data 

# Proteomic Analysis of *Rhizoctonia solani* Identifies Infection-specific, Redox Associated Proteins and Insight into Adaptation to Different Plant Hosts

## Supplemental Data

- Supplementary Table S1 (.pdf, 28 KB)
- Supplementary table S2 (.xlsx, 898 KB)
- Supplementary table S3 (.pdf, 18 KB)
- Supplementary table S4 (.xlsx, 50 KB)
- Supplementary table S5 (.xlsx, 32 KB)
- Supplementary table S6 (.xlsx, 57 KB)
- Supplementary table S7 (.pdf, 35 KB)
- Supplementary figure S1 (.pdf, 1.7 MB)
- Supplementary figure S2 (.pdf, 27 KB)
