# Supplementary material for: Proteomic Analysis of Rhizoctonia solani Identifies Infection-specific, Redox Associated Proteins and Insight into Adaptation to Different Plant Hosts
Source: Mol Cell Proteomics. 2016 Jan 25;15(4):1188–203. doi: 10.1074/mcp.M115.054502 (PMC4824849; doi:10.1074/mcp.M115.054502)
Supplement: Supplemental Data [file 10.1074_M115.054502_mcp.M115.054502-1.pdf]

Supplementary table S1: Primers used for QPCR analysis of gene expression

Gene	Primer	Primer
RSAG8G_00146	CCTTCACGACGAGGTTCG	CTGGAACTTGCTGCACCC
RSAG8G_00435	GTGCATCACAGCGTCTGG	GTGGAGTTCCAAGCCTGC
RSAG8G_00435	GTGCATCACAGCGTCTGG	GTGGAGTTCCAAGCCTGC
RSAG8G_00448	AAGGGTCAATTCGGAGCC	GATGCTGTGATTGCACCG
RSAG8G_01617	GGATGAGCTCCTTGCTGC	GCGTGATATCCACCTGGC
RSAG8G_02244	GCCAAGCTGGTCAAAACC	CATCAAAGATGGTCCCCG
RSAG8G_02343	TTTCACAATACGCTGCGG	CCATCTCCTCAAGCGGG
RSAG8G_02727	ACGGTCAAACCCAGAACG	AAGCACGGTGAAACGACC
RSAG8G_03246	GTGGACAAAATGCCCAGC	TCAACTTCGTCCAACAGGC
RSAG8G_03280	TAGCTCGCACTGTCCAAATG	AACGACCATGGGAAGCATAG
RSAG8G_03830	CCGCTCCTACTTTGCTCG	GCCACCAACCAACGTACC
RSAG8G_03874	CGGACATAGCTTGGGTGG	TGCCCTGAGCAAGCTACC
RSAG8G_04390	GACTTGGCTGGTCGATGC	CCGTTCGGGTACTTGTCG
RSAG8G_04560	CTGCCTCGCTTGTTCACC	CAAACTGGGCGCTTTCC
RSAG8G_05829	CTCACGTGGTTCTTCGCC	CATTGCACCGTCTCTCCC
RSAG8G_05942	TTGTTGGGCACTCTGACG	ACCACCAAGGTCATTGCC
RSAG8G_05942	TTGTTGGGCACTCTGACG	ACCACCAAGGTCATTGCC
RSAG8G_05942	TTGTTGGGCACTCTGACG	ACCACCAAGGTCATTGCC
RSAG8G_06044	CATATGGAAAGCCCGTGG	CGATGGTGTTGATCGTCG
RSAG8G_06853	ACCAGCTCCGTCAAATCG	GGCACAGAACAAATCGCC
RSAG8G_07318	ATCTCTGGAGGGTGTGCG	GACCTACCAATCACGCCG
RSAG8G_07380	TGAGCTTCCTCACCTGCG	CACCAAGGGTGACAACGG
RSAG8G_07433	GGTTTGAGGACGTTTGCG	TTGGAGTTGATGCCAGCC
RSAG8G_07433	GGTTTGAGGACGTTTGCG	TTGGAGTTGATGCCAGCC
RSAG8G_07489	TGCTCCTTCCGATAAGCG	CCCTCGTTCTGTTTTGCG
RSAG8G_07489	TGCTCCTTCCGATAAGCG	CCCTCGTTCTGTTTTGCG
RSAG8G_07504	GCAACGTCTGGTTCGAGG	GTTCGAGTTCACACCCGC
RSAG8G_07504	GCAACGTCTGGTTCGAGG	GTTCGAGTTCACACCCGC
RSAG8G_07875	AGGAACTGATGGATGCGG	TGGACTTGCTGTTGTCCG
RSAG8G_08039	ACGTCGGAGCTCACTTGG	TGTTAGAGCCGGAGGAGC
RSAG8G_09107	ATCCTGCTACCTTGGCCG	CAACTCGTTGACCCGAGC
RSAG8G_09107	ATCCTGCTACCTTGGCCG	CAACTCGTTGACCCGAGC
RSAG8G_09852	TTGTGAGGCGACTGGAGG	TCACTGGACGATCAGGGG
RSAG8G_10274	GATCAACTTTGGCAGCCC	AGATAAAGTGGCCGTCGC
RSAG8G_10341	CTCTTCTTGCCGGATTGG	AAGTACCTGCCACAGCGG
RSAG8G_11016	TGGAGAGACCTCCAACGC	CTGTGTTCCCTGGTTCGC
RSAG8G_11016	TGGAGAGACCTCCAACGC	CTGTGTTCCCTGGTTCGC
RSAG8G_11570	GCCGTCTTGGGAGAATCC	CGTGATCCAAGTCGGAGG
RSAG8G_11570	GCCGTCTTGGGAGAATCC	CGTGATCCAAGTCGGAGG
RSAG8G_12186	AATGCGGGTGATACTGGC	TCAGGAGGGCAGCTAACG
RSAG8G_13658	ATGCACGGACCTCTCTGG	TTTGAGCACCCAGGAAGC
RSAG8T_02576	CCCAGTGAAGAGTATGAGGGC	TCCTTGAGTGAAGTCTTCCGC

Supplementary table S1 continued.

Gene	Primer	Primer
RSAG8T_02776	CGACCACATCCTCTTTTGGGA	CCAAATGTGACCGCCAGTTTT
RSAG8T_03274	ACTGGAGTGCAGATTGTTCGT	TTTCGGTACCTTGGAACCTCG
RSAG8T_03407	GACGGTGTCTATTCCCTCGAG	AACGTGGAATTGTTTGGCTCG
RSAG8T_05194	TGCTGCCAACGTTTTCAACTA	GTAGTGCCGGATGAGATACCA
RSAG8T_05661	CGCGGCTTATTGAACGATCTG	ATGAACATGGCCTTCGACAGT
RSAG8T_06785	GCGCGGACATGATTTAATGCT	CAAACGGCGTCCAAAAGTTCT
RSAG8T_08263	TTGCTGAGATGTTGTTGCACG	AATAGACTGGAAAGGGCGACG
RSAG8T_11272	GACCTAGACCAACACGCTCAA	TCTCGTTCCATTCCAGGTGTG
Scaffold_12_2413_137178_137639_+	GCAAATTCGAGCAACTGGTGT	TGGAGAGTGTATGCGCCTTTT
Scaffold_197_1383_46180_46476_-	TGGGATTTGATGCTAACGGGT	GATTCTGCAAAGCGACAGTGG
Scaffold_205_243_14147_15250_+	AGCCTTCCCTAATACGACCCT	ATCAGGAGACGAGGTAGCAGT
Scaffold_280_1040_12924_13205_-	GCACGCACATCCAATAAACGG	CCTCGTCATGCCTTCTCTTCA
Scaffold_58_2876_132842_133237_+	ATCTACAAGGTTCTCGACGCC	CGAATCGGTCAGAACACTTGC
Scaffold_8_27139_72994_73251_-	AACCTGAACCTACCGCGTTC	CAGCTCACAAGTCAACTGGG
Scaffold_8_27144_72661_72981_-	GGTGTGTCCTCGTCTCTCTTG	AGGACCATAGCAAAGGAAGGTG
Scaffold_81_261_10003_10203_+	TATTGGAGGCGACAACTACCG	CGACTTCCTCCTTCGTCACAA
Beta-catenin (Control)	TGGATGAGGACGAGGAGC	GGCTTGTAGTTGCTGGCG
C3HC4 zinc finger (Control)	TACCCACAGAGCCTTCGG	GACATCAACACCTCCGGC
ITS	AGAGTTGGTTGTAGCTGGTCC	CCGTTGTTGAAACTTAGTATTAGA
Ribosomal protein L14 (Control)	ATGCTTGGTCTCAAAGCG	ACTTTGATCGCTGTTGCC
RNA recognition protein (Control)	AGCCCGAATCTGTTCACG	GGTCGTTGAAACCCATGC
