# Supplementary material for: Proteomic Analysis of Rhizoctonia solani Identifies Infection-specific, Redox Associated Proteins and Insight into Adaptation to Different Plant Hosts
Source: Mol Cell Proteomics. 2016 Jan 25;15(4):1188–203. doi: 10.1074/mcp.M115.054502 (PMC4824849; doi:10.1074/mcp.M115.054502)
Supplement: Supplemental Data [file 10.1074_M115.054502_mcp.M115.054502-3.pdf]

Supplementary table S3: Spectra mapping to different sequences in the *R. solani* and wheat databases..

Treatment	Tissue	Time point	R.solani Protein accession number	R.solani Peptide sequence	Modifications identified by spectrum	Number of unique peptides	Number of total spectra	Spectrum name	SEQUEST XCorr score	Mascot Ion score	Wheat entry found	Wheat Peptide sequence	SEQUEST XCorr score	Mascot Ion score	Comment	
Infection	Membrane	Early (3 dpi)	RSAG8_03810	NSPAIIFIDEIDSIAPK		23	42	File10594 Spectrum31123 scans: 33809	4.61	74.5	Traes_4DL_DBA53B48C.1	NAPSIIFIDEIDSIAPK	4.35	52.9	3AA shuffled (SPA vs APS)	
Infection	Membrane	Early (3 dpi)	RSAG8_03810	NSPAIIFIDEIDSIAPK		23	42	File10594 Spectrum31433 scans: 34140	4.34	60.4	Traes_4DL_DBA53B48C.1	NAPSIIFIDEIDSIAPK	4.03	33.5	3AA shuffled (SPA vs APS)	
Infection	Membrane	Early (3 dpi)	RSAG8_03810	NSPAIIFIDEIDSIAPK		23	42	File10594 Spectrum31969 scans: 34726	3.68	34.2	Traes_4DL_DBA53B48C.1	NAPSIIFIDEIDSIAPK	3.41	31	3AA shuffled (SPA vs APS)	
Infection	Membrane	Late (7 dpi)	RSAG8_03810	NSPAIIFIDEIDSIAPK		20	33	File10602 Spectrum30430 scans: 32990	4.86	93.4	Traes_4DL_DBA53B48C.1	NAPSIIFIDEIDSIAPK	4.31	58.4	3AA shuffled (SPA vs APS)	
Infection	Membrane	Early (3 dpi)	RSAG8_03810	VLNQILTEMDGMNAK	Oxidation (+16)	23	42	File10594 Spectrum14006 scans: 15653	3.41	46.9	Traes_4DL_DBA53B48C.1	VLNQLLTEMDGMNAK	3.41	38.1	Ambiguous AA (I vs L)	
Infection	Membrane	Early (3 dpi)	RSAG8_03810	VLNQILTEMDGMNAK		23	42	File10594 Spectrum17448 scans: 19269	4.89	60	Traes_4DL_DBA53B48C.1	VLNQLLTEMDGMNAK	4.89	47.7	Ambiguous AA (I vs L)	
Infection	Membrane	Late (7 dpi)	RSAG8_03810	VLNQILTEMDGMNAK	Oxidation (+16)	20	33	File10602 Spectrum12703 scans: 14375	4.21	70.3	Traes_4DL_DBA53B48C.1	VLNQLLTEMDGMNAK	4.21	70.3	Ambiguous AA (I vs L)	
Infection	Membrane	Late (7 dpi)	RSAG8_03810	VLNQILTEMDGMNAK	Oxidation (+16)	20	33	File10602 Spectrum8925 scans: 10408	4.47	63	Traes_4DL_DBA53B48C.1	VLNQLLTEMDGMNAK	4.47	54.2	Ambiguous AA (I vs L)	
Infection	Culture filtrate	Late (7 dpi)	RSAG8_05268	YPNSGDGPYAGHIMVVSEQK	Deamidated (+1), Deamidated (+1)	3	73	File10590 Spectrum10668 scans: 11961	2.62	17.5	Traes_1AL_E96C0662D.2	YCSILGTATGGNLDCYTQR	4.26	37.1		
Infection	Membrane	Early (3 dpi)	RSAG8_07749	IPIFSAAGLPHNEIAAQICR	Carbamidomethyl (+57)	5	5	File10594 Spectrum19027 scans: 20927	4.31	61	tr|W5DET9|W5DET9_WHEAT	IPLFSAAGLPHNEIAAQICR	4.31	45.4	Ambiguous AA (I vs L)	
Infection	Membrane	Late (7 dpi)	RSAG8_07749	IPIFSAAGLPHNEIAAQICR	Carbamidomethyl (+57)	10	16	File10602 Spectrum17571 scans: 19487	4.2	46.4	tr|W5DET9|W5DET9_WHEAT	IPLFSAAGLPHNEIAAQICR	4.2	38.2	Ambiguous AA (I vs L)	
