# Supplementary material for: Proteomic Analysis of Rhizoctonia solani Identifies Infection-specific, Redox Associated Proteins and Insight into Adaptation to Different Plant Hosts
Source: Mol Cell Proteomics. 2016 Jan 25;15(4):1188–203. doi: 10.1074/mcp.M115.054502 (PMC4824849; doi:10.1074/mcp.M115.054502)
Supplement: Supplemental Data [file 10.1074_M115.054502_mcp.M115.054502-7.pdf]

Supplementary table S7. The number of proteins putatively associated with carbohydrate active enzyme (CAZy) categories from the culture filtrate samples.

Family	Count	Brief description
AA1	4	Laccases, ferroxidases and laccase-like multicopper oxidases
AA10	2	Chitin and cellulose cleavage
AA2	1	Class II lignin-modifying peroxidases
AA3	2	Glucose-methanol-choline (GMC) oxidoreductases
AA3|AA8	2	GMC oxidoreductases and Iron reductase domains
AA5	5	Glyoxal oxidases or galactose oxidases
AA7	1	Glucooligosaccharide oxidases
AA8	1	Iron reductase domain
AA9	5	Cellulose cleavage
AA9|CBM1	3	Cellulose-binding module
CBM12|GH18	1	Chitin-binding module, chitinase
CBM12|GH19|CBM5	1	Chitin-binding module, chitinase
CBM13	15	Carbohydrate-binding
CBM14	1	Chitin-binding module
CBM20	1	Starch-binding domain
CBM20|GH15	5	Starch-binding domain
CBM63	3	Cellulose binding module
CE10	3	Esterase activity
CE4	13	Deacetylase/esterase activity
GH103	2	Peptidoglycan lytic transglycosylase
GH13	1	Amylase
GH16	7	Glycoside Hydrolase
GH18	3	chitinases and non-catalytic proteins such as xylanase inhibitor
GH2	4	Glycoside Hydrolase
GH31	1	Glycoside Hydrolase
GH38	1	Mannosidase
GH43	2	Glycoside Hydrolase
GH47	2	Mannosidase
GH5	3	Glycoside Hydrolase
GH7	3	Cellobiohydrolases
GH72|CBM43	3	β-1,3-glucanosyltransglycosylase, β-1,3-glucan binding module
GH76	1	α-1,6-mannanase
GH88	1	d-4,5-unsaturated β-glucuronyl hydrolase
GT2	1	Includes cellulose synthase and chitin synthase
GT34	1	Glycosyltransferase
GT35	2	Glycogen or starch phosphorylase
GT4	1	Glycosyltransferase
GT41	1	Glycosyltransferase
GT66	1	Protein glycotransferase, utilize a lipid-diphospho-oligosaccharide as the donor
GT74	1	α-1,2-L-fucosyltransferase
GT90	2	Glycosyltransferase
PL3	1	Pectate lyase
PL8	2	Hyaluronate lyase
