# Supplementary material for: Proteomic Analysis of Rhizoctonia solani Identifies Infection-specific, Redox Associated Proteins and Insight into Adaptation to Different Plant Hosts
Source: Mol Cell Proteomics. 2016 Jan 25;15(4):1188–203. doi: 10.1074/mcp.M115.054502 (PMC4824849; doi:10.1074/mcp.M115.054502)
Supplement: Supplemental Data [file 10.1074_M115.054502_mcp.M115.054502-8.pdf]

A.

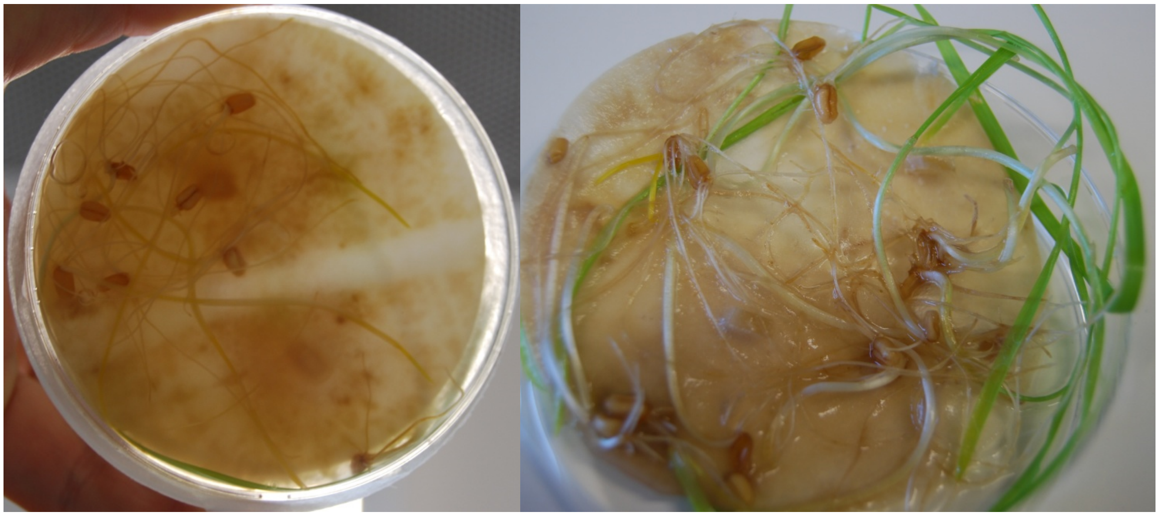

3 dpi 7 dpi

B.

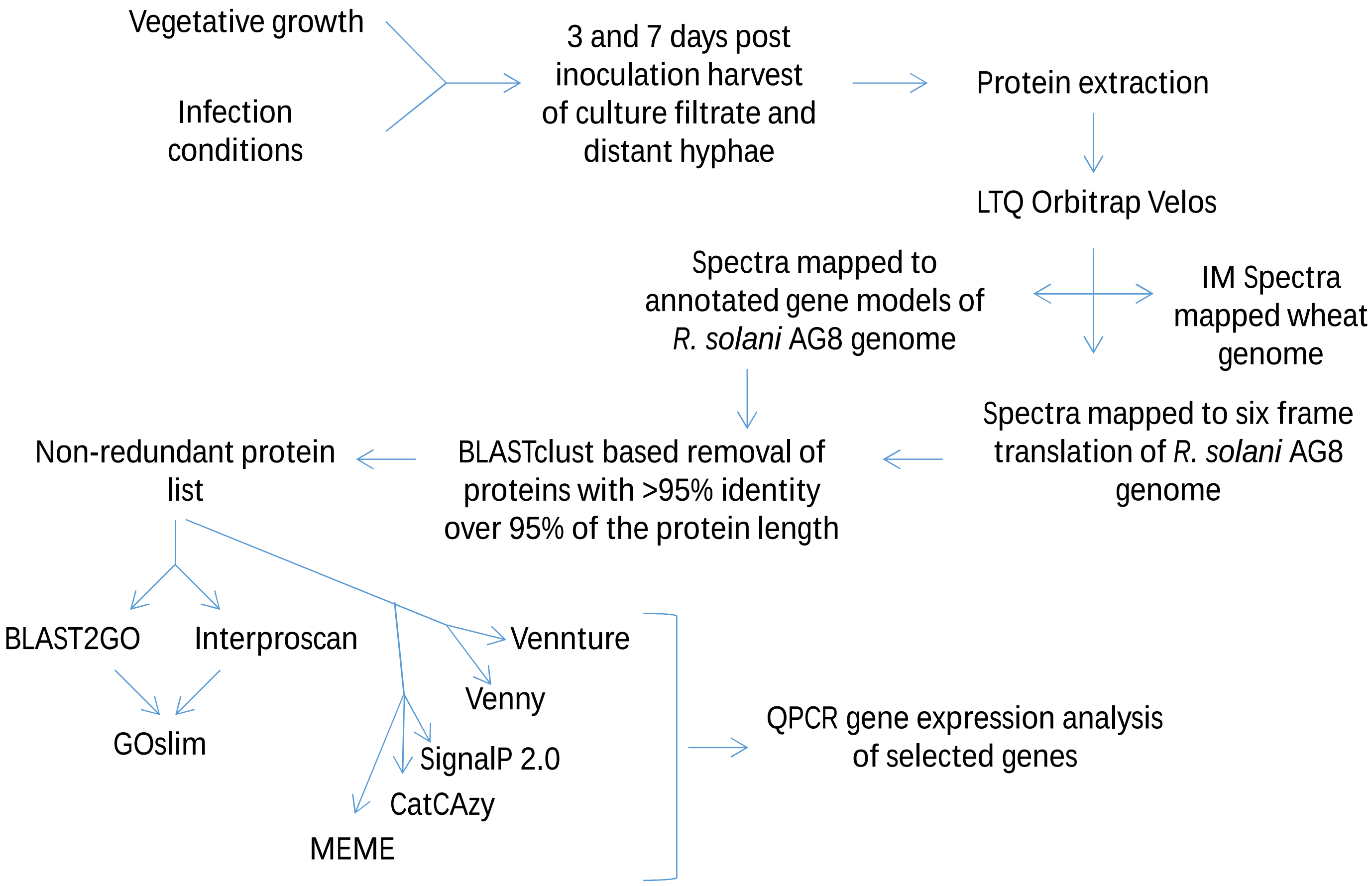

Supplementary figure S1. A, Infection conditions at time of harvest at 3 days post inoculation and 7 days post inoculation. B, Workflow diagram for experimental procedures
