# Supplementary material for: Proteomic Analysis of Rhizoctonia solani Identifies Infection-specific, Redox Associated Proteins and Insight into Adaptation to Different Plant Hosts
Source: Mol Cell Proteomics. 2016 Jan 25;15(4):1188–203. doi: 10.1074/mcp.M115.054502 (PMC4824849; doi:10.1074/mcp.M115.054502)
Supplement: Supplemental Data [file 10.1074_M115.054502_mcp.M115.054502-9.pdf]

A. Cellular location of proteins from mycelia samples

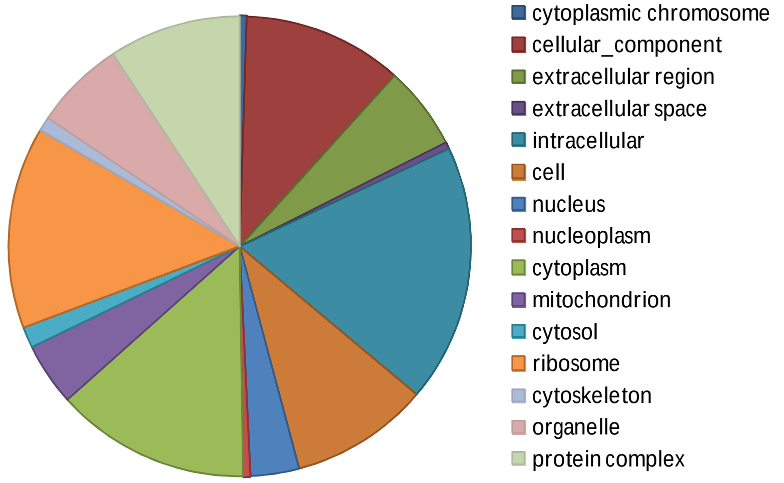

B. Cellular location of proteins from culture filtrate samples

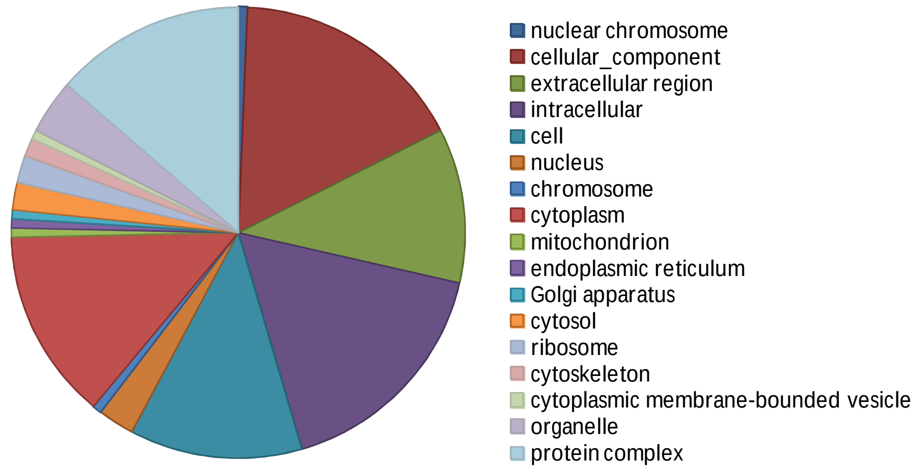

C. Cellular location of proteins from membrane samples

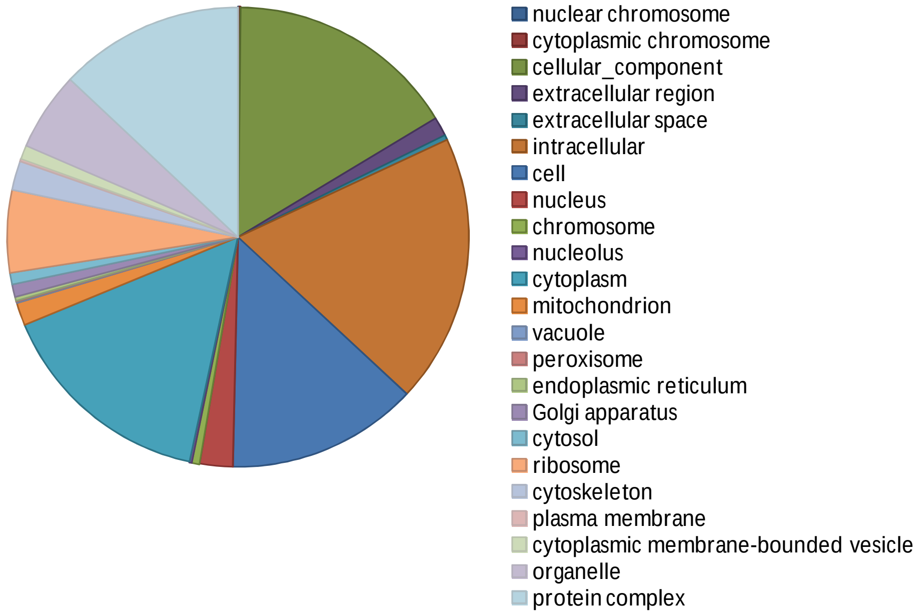

Supplementary figure S2. Representation of the proportions of cellular location annotations for A, mycelia samples, B, culture filtrate samples and C, membrane samples.
